# Conventional, Speed Sintering and High-Speed Sintering of Zirconia: A Systematic Review of the Current Status of Applications in Dentistry with a Focus on Precision, Mechanical and Optical Parameters

**DOI:** 10.3390/jcm11164892

**Published:** 2022-08-20

**Authors:** Nadin Al-Haj Husain, Mutlu Özcan, Nataliya Dydyk, Tim Joda

**Affiliations:** 1 Department of Reconstructive Dentistry and Gerodontology, School of Dental Medicine, University of Bern, 3010 Bern, Switzerland; 2 Division of Dental Biomaterials, Clinic of Reconstructive Dentistry, Center of Dental Medicine, University of Zurich, 8032 Zurich, Switzerland; 3 Department of Prosthetic Dentistry, Danylo Halytsky Lviv National Medical Universtiy, 79010 Lviv, Ukraine; 4 Clinic of Reconstructive Dentistry, Center of Dental Medicine, University of Zurich, 8032 Zurich, Switzerland; 5 Department of Reconstructive Dentistry, University Center for Dental Medicine Basel, University of Basel, 4058 Basel, Switzerland

**Keywords:** aesthetics, all-ceramic, clinical outcome, cost efficacy, dental, dental materials, firing, mechanical, post-processing, pre-processing, prosthetic dentistry, sintering, speed sintering, systematic review, technical, time efficacy, zirconia, zirconium dioxide

## Abstract

The aim of this systematic review was to provide an overview of the technical and clinical outcomes of conventional, speed sintering and high-speed sintering protocols of zirconia in the dental field. Data on precision, mechanical and optical parameters were evaluated and related to the clinical performance of zirconia ceramic. The PICOS search strategy was applied using MEDLINE to search for in vitro and in vivo studies using MeSH Terms by two reviewers. Of 66 potentially relevant studies, 5 full text articles were selected and 10 were further retrieved through a manual search. All 15 studies included in the systematic review were in vitro studies. Mechanical, precision and optical properties (marginal and internal fit, fracture strength and modulus, wear, translucency and opalescence, aging resistance/hydrothermal aging) were evaluated regarding 3-, 4- and 5-YTZP zirconia material and conventional, high- and high-speed sintering protocols. Mechanical and precision results were similar or better when speed or high-speed sintering methods were used for 3-, 4- and 5-YTZP zirconia. Translucency is usually reduced when 3 Y-TZP is used with speed sintering methods. All types of zirconia using the sintering procedures performed mechanically better compared to lithium disilicate glass ceramics but glass ceramics showed better results regarding translucency.

## 1. Introduction

Tooth loss in humans may lead to physical and mental impairment affecting self-esteem and quality of life [1]. In order to maintain a high quality of life, missing teeth or hard tissues can be replaced using fixed dental prostheses (FDPs). Commonly used materials for tooth- and implant-borne FDPs are metal-ceramic or all-ceramic materials [2]. All-ceramic restorations can be veneered using a glassy matrix ceramic system or used in their monolithic state [3]. Zirconia ceramics containing yttria-stabilized tetragonal zirconia polycrystals (Y-TZP) exhibit the highest mechanical (fracture toughness and flexural strength), good biological (biocompatibility) and promising aesthetic properties [4,5]. FDPs, resin-bonded FDPs and full-arch dental prostheses made of zirconia show similar longevity when compared to established metal-ceramic restorations [6,7]. 

Zirconia restorations are produced using computer-aided design and computer-aided manufacturing (CAD/CAM) technologies in either a dental laboratory or clinical setting. Three different manufacturing routes are available, the clinical in-office chairside or the laboratory setting either using in-lab systems or centralized milling centers [8,9]. 

To process zirconia with lab- and chairside technologies, it can be milled using two types of CAD/CAM systems: Hard machining of sintered blocks or soft machining of partially sintered blocks. There are different indications and procedures involved in these two methods. While hard milling is used for dental implant and implant abutment fabrication, it is considered as difficult and expensive due to its high strength and hardness, necessitating longer milling time and causing higher wear of the milling tools. The sintering of zirconia is different than that of other types of dental ceramics, and traditional furnaces may not be able to sinter it to fulfil the requirements for intraoral use. Fully sintered zirconia is approximately twice as dense as pre-sintered zirconia, requiring nearly 45 min of milling and a new tool per restoration [10].

Due to the difficulties involved, soft milling is used for single- and multi-unit FDPs in a pre-sintered porous state. However, after soft milling, zirconia has to undergo additional sintering procedures to achieve full density, requiring a 6–8 h procedure including heating, cooling and dwelling time. While hard milling can be carried out at a 1:1 ratio, needing further sintering, specimens machined by soft milling should be milled to a 25% larger size to compensate for sintering shrinkage [11]. 

Compared to labside procedures, a chairside protocol allows the clinician to design and manufacture the dental prosthesis in the dental practice for single-visit restorative treatment. However, soft milled zirconia restorations could not be considered as a single visit treatment option because of the need for a conventional post-milling sintering procedure lasting several hours and requiring a furnace reaching 1500 °C [12]. Recently, chairside zirconia milling speed (60–120 min) and high-speed (10 min) sintering protocols for partially sintered zirconia have been developed using novel induction furnaces [12].

Conventional furnaces contain heating resistance elements, molybdenum disilicate, where heat is generated through an electric current passing through the resistor and heating the surrounding air. Resistance elements limit the heating rates of the furnace to 40 to 70 °C per min [13]. In contrast, an induction furnace uses electromagnetic induction to apply heat or passes an electric current through an object by an alternating magnetic field [13]. Zirconia restorations are placed in a susceptor body through which the induction furnace generates a current by an alternating magnetic field in a copper tube-wrapped susceptor body. First the zirconia will be heated indirectly by the susceptor body, and later directly through the magnetic field, allowing higher heating rates. The sintering parameters except for the duration are usually not provided by the manufacturers. Some studies state that the final temperature in induction furnaces must be higher and is achieved faster to provide sufficient sintering [14], while others argue that high-speed sintering protocols, including the 10 min holding time at a 1570–1590 °C heating temperature and a heating rate of 50–100 °C per min, are sufficient for sintering.

The sintering procedure, and especially the sintering time and temperature in addition to hydrothermal aging may affect the grain structure, and is thereby associated with the mechanical and optical properties of zirconia. The microstructure of zirconia consists of small crystalline structures surrounded by grain boundaries and is available in different configurations: yttria-stabilized tetragonal zirconia polycrystal with different Yttria mol percentages (3Y-TZP, 4Y-TZP and 5Y- TZP). The 3-YTZP zirconia is composed of tetragonal phase, while the 4 or 5 mol% yttria zirconia contain a significant percentage of cubic grains. Data on the influence of speed and high-speed sintering protocols on precision, mechanical and optical clinical performance of zirconia are lacking. Therefore, the aim of this systematic review was to analyze the influence of conventional, speed and high-speed sintering procedures on the clinical precision, mechanical and optical parameters of zirconia.

## 2. Materials and Methods

### 2.1. Search Strategy

Following a preliminary search, a PICO research question was defined: “For sintering zirconia ceramics, is speed sintering comparable to conventional sintering in terms of mechanical characteristics, quality fit and volume stability, aesthetics, pre- and postprocessing, and economics related to time-/cost-efficiency?” The search focused on material choice (zirconia); sintering protocol (conventional, speed and high-speed sintering); indication (implant and tooth-borne single restoration unit and reconstruction design (partial crown single units vs. crown)) and mechanical, clinical and economic outcomes (including pre- and post-processing). 

The PICO research question was then chosen as follows: *P-*population: tooth and implant-borne partial or full zirconia restorations; I-intervention: speed and high-speed sintering; C-control: conventionally manufactured/produced restorations (sintering, firing); O-outcome: mechanical properties; mechanical stability, flexural strength, microstructure, grain size, translucency, volume stability, internal fit, marginal fit, clinical behavior, adverse event and aesthetics; S-study designs: in vivo and in vitro studies.

The following search terms, MeSH terms and combinations were used in the Pubmed search: (((dental crowns [MeSH]) OR (full crown) OR (partial crown) OR (table top)) AND((zirconia [MeSH]) OR (ZrO2) OR (zirconium dioxide)) AND (((speed sintering) OR (high-speed sintering)) OR ((sintering) OR (firing))) AND(((mechanical properties) OR (mechanical stability) OR (flexural strength) OR (microstructure) OR (grain size) OR (translucency)) OR ((volume stability) OR (internal fit) OR (marginal fit)) OR ((clinical behavior) OR (adverse event) OR (esthetics)) OR ((economics [MeSH Terms]) OR (time-efficiency) OR (cost effectiveness) OR (cost analysis)))). The search strategy is presented in Table 1. 

The following terms were used in the EMBASE search: (‘dental crowns’/exp OR ‘full crown’/exp OR ‘partial crown’/exp OR ‘table top’) AND (‘zirconia’ OR ‘ZrO2’ OR ‘zirconium dioxide’) AND ((speed sintering’ OR ‘high-speed sintering’) OR (‘sintering’ OR ‘firing’)) AND ((‘mechanical properties’ OR ‘mechanical stability’ OR ‘flexural strength’ OR ‘microstructure’ OR ‘grain size’ OR ‘translucency’) OR (‘volume stability’ OR ‘internal fit’ OR ‘marginal fit’) OR (‘clinical behavior’ OR ‘adverse event’ OR ‘esthetics’) OR (‘economics’ OR ‘time-efficiency’ OR ‘cost effectiveness’ OR ‘cost analysis’)) NOT [medline]/lim AND [embase]/lim.

The following terms were used in the Web of Science and IADR abstracts search: ((((((((dental crowns [MeSH]) OR (full crown) OR (partial crown) OR (table top)) AND ((zirconia [MeSH]) OR (ZrO2) OR (zirconium dioxide [MeSH])) AND (((speed sintering) OR (high-speed sintering)) OR ((sintering [MeSH]) OR (firing [MeSH]))) AND (((mechanical properties) OR (mechanical stability) OR (flexural strength) OR (microstructure) OR (grain size) OR (translucency)) OR ((volume stability) OR (internal fit) OR (marginal fit)) OR ((clinical behavior) OR (adverse event) OR (esthetics)) OR ((economics) OR (time-efficiency) OR (cost effectiveness) OR (cost analysis))).

### 2.2. Information Sources

The electronic databases Web of Science (ISI—Web of Knowledge), EMBASE and PubMed MEDLINE, including IADR abstracts and Google Scholar were used for the systematic electronic literature search until 8 December 2020. In vitro and in vivo articles in English were selected. In vivo studies included were those performed on human species and published in dental or medical journals. 

### 2.3. Study Selection and Eligibility Criteria

**Figure 1:**
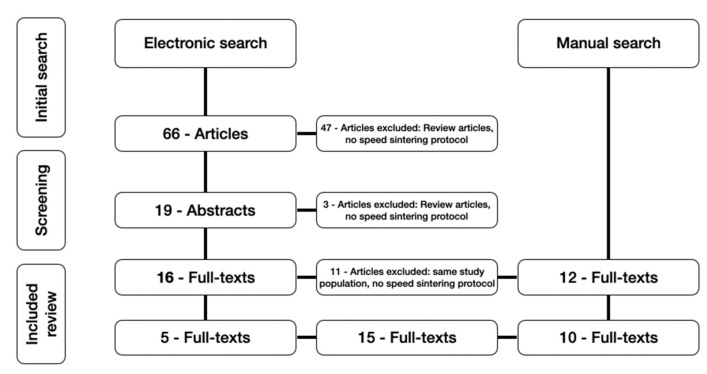
Systematic search results illustrated as a Flow diagram.

Two reviewers (N.A. and T.J.) independently conducted an electronic literature search and selection of the studies. Both evaluated titles and abstracts of the retrieved studies and disagreements were discussed. Out of 66 studies, 5 were selected in full texts and the inclusion of the studies was made according to the chosen inclusion criteria (Figure 1).

The chosen inclusion criteria were the following: (1) in vitro and in vivo studies published prior to December 2021; (2) studies evaluating dental zirconia materials; (3) studies in English. 

The exclusion criteria were: (1) studies that did not compare two or more sintering protocols or pre- and post-speed sintering or (2) evaluated materials other than zirconia. 

### 2.4. Data Extraction and Collection

All data were screened, and titles and abstracts were extracted and evaluated according to the inclusion criteria. Selected abstracts were obtained as full texts. In cases of compliance with the inclusion criteria, full texts were retrieved and the following data from the included articles were obtained for further analysis: demographic information (title, authors, journal and year), study specific parameters (study type, number of specimens), materials tested (type and commercial name, manufacturing technique), sintering protocol (method, temperature and duration), mechanical, optical and technical parameters (fracture strength and modulus, wear, translucency and opalescence, microstructure, chemical composition, marginal and internal fit, dimensional change and aging resistance/ hydrothermal aging) and outcomes. 

## 3. Results

### 3.1. Study Selection

A total of 19 Studies were selected for full text analysis out of 66 potentially relevant ones, and 5 were finally included in the systematic review. A further 10 studies were retrieved through manual search. All 15 studies included in the systematic review were in vitro studies [15,16,17,18,19,20,21,22,23,24,25,26,27,28,29].

### 3.2. Study Characteristics

Included studies and their characteristics are shown in Table 2. They were published between 2015 and 2021. A total of 15 studies including 1822 specimens were evaluated. The excluded studies either did not meet the inclusion criteria, were reviews, not in peer-reviewed journals, not written in the English language or did not compare different sintering parameters or methods of zirconia sintering and their effect on precision, mechanical and optical properties or microstructure and phase composition.

All studies included were in vitro studies. The materials included were 3–5 mol% Y-TZP monolithic zirconia and lithium disilicate. All specimens were either milled using milling machines or cut using diamond wire saws. The sintering was performed according to three different sintering protocols (conventional, speed and high-speed sintering) using sintering or high-temperature sintering furnaces.

The following mechanical, optical and technical parameters were assessed in the retrieved publications: fracture strength and modulus, wear, translucency and opalescence, microstructure, chemical composition, marginal and internal fit, dimensional change and aging resistance/ hydrothermal aging.

## 4. Discussion

The scientific utility of this systematic review was the evaluation of in vitro and in vivo data on tooth and implant-borne partial or full zirconia restorations using different sintering protocols (conventional, speed and high-speed) with varying sintering temperatures and durations. No clinical investigations were found. In vitro data focused on precision, mechanical and optical parameters, such as marginal and internal fit, fracture strength and modulus, wear, translucency and opalescence, microstructure, chemical composition, dimensional change and aging resistance/ hydrothermal aging. Altering the sintering duration and temperature as well as stabilizer content determines microstructure and grain size and influences their precision, mechanical and optical properties and aging resistance [30]. 

In a changing dental field undergoing digital transformation, dentists and patients are facing major social changes, where the aesthetic and economic performance indicators (EPI) [31] become relevant factors to be considered when planning a dental rehabilitation. Dentists aim to keep up with the high competitiveness by creating unique selling points and keeping the value chain in-house. For the patient, functional rehabilitation is assumed and the main focus is on minimal- or non-invasiveness, aesthetics, costs and time [31].

For these reasons, the dental industry has reacted and introduced the chairside trend in indirect CAD/CAM reconstructions including speed and high-speed sintering procedures, which enable the sintering of zirconia restorations in minutes, enhancing the efficacy of single-visit treatments. However, various technical and clinical parameters must be investigated regarding rapid sintering protocols.

Precise marginal fit of restorations supports periodontal health, as properly fitted restorations prevent plaque accumulation compared to non-precise restorations. Studies by Rezende et al., Ahmed et al., Elisa Kauling et al. and Nakamura et al. evaluated the fit precision of speed sintered zirconia-based restorations compared to conventional sintered ones [16,17,18,27]. Ahmed et al. [16] evaluated the margin fit using optical microscopy and reported that fast sintering resulted in greater marginal discrepancies of monolithic zirconia crowns than with the standard sintering protocol. However, the study carried out by Elisa Kauling et al. showed after speed and conventional sintering of 3-unit zirconia FDPs using the replica technique a slightly superior fit in the marginal and occlusal areas of zirconia restorations sintered by speed processes compared to conventionally sintered ones [27]. When single abutment teeth were investigated, speed sintering showed a significantly better fit compared to the conventional sintering in premolars at the marginal gap and occlusal surface, and in molars at the occlusal surface. This was explained by the higher predictability of the shrinkage of zirconia during speed-sintering processes. However, all values were within the clinically acceptable range. Nakamura et al. [17], on the other hand, found no significance in the marginal gap high-speed sintered monolithic zirconia crowns compared to conventionally fabricated ones after aging. The mean internal gap, however, was significantly higher in the speed sintered restorations. This difference was attributed to software selection used to manufacture the restorations. Earlier, Rezende et al. investigated the internal fit and dimensional sintering changes of Y-TZP coping by micro computed tomography (Micro-CT). They observed a not evenly distributed sintering shrinkage. The internal fit discrepancy was also not even, with the highest fit in the occlusal regions [27]. However, the marginal fit values were in the clinically acceptable range. A statistical significance was observed between shrinkage values reported by the corresponding manufacturer and the experimental results. Furthermore, varying geometrical specimens and the tested copings resulted in statistically significant sintering shrinkage. In conclusion, it can be stated that regarding precision further investigations are needed, as the studies show contradicting results. However, regardless of the sintering protocol used, the precision is in a clinically acceptable range.

With regard to mechanical properties, several studies emphasized similar mechanical values of zirconia obtained for conventional and speed sintering [15,17,21,25,27,28]. Soult et al. found comparable strength properties (flexural strength and modulus) of zirconia groups manufactured in high-speed or conventional furnaces [15]. Even after low-temperature degradation under accelerated aging conditions, which is equivalent to decades of intraoral use, monolithic zirconia crowns did not show any fracture load differences under conventional and high-speed sintering conditions [17]. Cokic et al. further investigated the composition, physical and mechanical properties of 5Y-PSZ and 3Y-TZP zirconia materials, such as the microstructure, density, hardness, fracture toughness and flexural strength under aging conditions, and could not find any differences between conventional and speed sintering protocols [21]. The 5Y-PSZ showed lower flexural strength and fracture toughness compared to 3Y-TZP irrespective of the sintering method [21]. The fracture load of 3-unit FDPs of monolithic zirconia also showed similar results under hydrothermal aging and conventional and speed sintering protocols [27]. Similarly, the fracture load and two-body wear were comparable in color-gradient multilayered zirconia [28], and flexural strength of manually colored 4Y-TZP [25] when varying sintering protocols were used. However, the shortened sintering procedures impaired aesthetic characteristics [25]. 

Contrary to previous findings, one study observed less two-body wear of 4Y-TZP zirconia when high-speed sintering was applied [19]. A higher fracture load was also reported using high-speed sintering methods, which was in agreement with the findings of Jansen et al., reporting higher flexural strength for 3Y-TZP (1590 °C compared to 1570 °C) [24,29]. Lawson et al. described less strength resistance in two 5-YTZP materials after high-speed sintering protocols, whereas the strength resistance of 3-YTZP zirconia remained unchanged [23]. Structural changes in the form of micropits causing a scratched steatite surface were observed more frequently in speed (1510 °C) and super-speed (1580 °C) sintering protocols [20] compared to conventional ones. 

As for the optical performance, the light transmissive properties of dental zirconia materials have been experimentally studied in most studies included in this review. Manufacturers tried to improve the translucency of 3Y-TZP zirconia by reducing the content of alumina, controlling its grain size and manipulating its density. However, light transmission still remains low for 3Y-TZP after conventional sintering. Reducing the size of the tetragonal grains might be an effective approach to increasing translucency in 3Y-TZP zirconia materials. When high-speed and conventional sintering procedures were compared, a smaller grain size was obtained using high-speed sintering procedures [15].

The effect of higher temperatures and shorter duration of sintering on the translucency of these materials with comparison to 4Y-TZP was investigated by Jansen et al. [24], where the translucency of two types of 3Y-TZPs with differing alumina content in Ceramill ZI (0.25%), Zolid (0.05%) and 4Y-TZP Zolid HT+ was evaluated using five different thicknesses ranging between 1 and 3 mm. It was observed that translucency decreased with increasing material thickness and that higher translucency was obtained using conventional sintering for Zolid, Zolid HT+ and ZI for thicknesses between 2.5 and 3 mm. Furthermore, the translucency was reduced especially after high-speed sintering of 3Y-TZP with 0.05 wt% Al_2_O_3_ and 4Y-TZP. These results were partially confirmed by Lawson et al. [23], stating that zirconia materials are affected differently using high-speed sintering protocols. Two (Prettau Anterior and Zpex Smile) out of three tested zirconia materials became less translucent and experienced large grain growth (from 1.24 to 4.11 μm) and porosities formation, leading to a reduction of the translucency. The zirconia material Katana STML remained unchanged. The changes in grain size were attributed to increased temperature and heating rates. One study explained the lower translucency after high-speed sintering in novel 3-YTZP materials (Zolid, Zolid HT+) with less Al_2_O_3_ as a result of higher particle density, reduced pore spaces during phase transformation and grain growth caused by higher temperatures and shorter sintering times. On the contrary, earlier 3Y-TZP materials achieve higher translucency when high sintering temperatures are used. 

To achieve higher translucency, attempts were made by producing partially stabilized 4Y- and 5Y-TZP materials with high nonbirefringent cubic phase content. A microstructure with a grain size under 100 nm without defects and pores was expected to allow light transmission without scattering. Speed and super-speed sintering methods producing dense and ultrafine Y-TZP grains by preventing their growth are therefore used to achieve high translucency [22]. Kaizer et al. confirmed this finding, as a better translucency performance of glazed monolithic molar crowns was achieved when super-speed sintering procedure was conducted compared to the conventional and speed sintering protocol [20]. These findings were supported further by in vitro studies of Soult et al. [15], who concluded that a high-speed induction furnace was capable of sintering full-contour 3Y-TZP zirconia specimens in substantially less time (26.2 min) than that needed for a conventional convection furnace (4.3 h), resulting in a significant grain size decrease with non-significant change in translucency or opalescence, although the mean translucency parameter was greater with higher sintering speeds. Cokic et al. [21] measured the translucency parameter of the zirconia ceramics (3Y-TZP and 5Y-PSZ) sintered with different speed protocols before and after hydrothermal aging. The 5Y-PSZ materials showed higher translucency compared to 3Y-TZO ceramics, which was not affected by varying sintering protocols. The 3Y-TZP, however, showed lower translucency in combination with speed-sintering. Hydrothermal aging for 60 h decreased only the translucency of the 3Y-TZP zirconia materials. 

Various studies have revealed the higher translucency of lithium disilicate ceramics compared to all investigated zirconia ceramics [21,23,28]. The translucency increased with higher Y-TZP contents (5Y-TZP > 4Y-TZP > 3-YTZP). Michailova et al. 2020 [28] evaluated strength-gradient and color-gradient multilayered zirconia sintered materials regarding translucency using conventional and high-speed protocols. Differences were observed within the layers of multilayered zirconia, with enamel layers showing higher translucency compared to transition and dentin layers. In the color-gradient zirconia, this could be explained by the color pigments in the body (dentin) area. For the strength-gradient zirconia, this difference was higher and is related to the different classes of zirconia (5Y-TZP in enamel layer and 3Y-TZP in dentin layer). 

As different techniques of zirconia materials coloring are currently used for matching zirconia with natural dentition, the implementation of metallic pigments to the zirconia powder prior or after milling or pressing, and immersion into coloring liquids, might also have an impact on the optical properties of zirconia. The effect of pigments and liquid on translucency and color matching were evaluated with emphasis on sintering time and effects of low-temperature degradation, also known as hydrothermal aging [25]. The authors reported that sintering time might influence manually colored zirconia materials, while aging decreased the translucency of all materials tested. The specimens did not reach the intended shade and appeared opaque and dull. This observation was attributed to the shortened sintering time, not allowing the diffusion of the color pigments into the microstructure, implying incomplete sintering. Pre-shaded high-speed sintered groups did not show these effects, as pre-sintering burns out the binding agent and activates the color pigments. Therefore, pre-shaded high-speed sintered 4Y-TZP resulted in lower translucency but higher color stability with less translucency decreases over time compared to conventionally sintered, manually colored zirconia. 

## 5. Conclusions

Based on this review, where only in vitro studies were found, mechanical, precision and translucency values were within the clinically acceptable range using all 3-, 4- and 5-YTZP zirconia. 

Mechanical and precision results were similar or higher when speed or high-speed sintering methods are used for 3-, 4- and 5-YTZP zirconia, and can be recommended for more cost- and time-efficient digital workflows in daily clinical practice.

Translucency is usually reduced when 3 Y-TZP is used with speed sintering methods. All types of zirconia using the sintering procedures performed mechanically better compared to lithium disilicate glass ceramics. However, glass ceramics showed better results regarding translucency.

## Figures and Tables

**Table 1:** PICO search strategy.

Focused Question (PICO)	For the Sintering of Zirconia, is Speed and High-Speed Sintering Comparable to Conventional Sintering in Terms of Mechanical Characteristics, Quality Fit and Volume Stability, Esthetics, Pre- and Post-Processing, and Economics Related to Time-/Cost-Efficiency?	
**Search strategy**	**Population**	*Single-unit tooth-borne and/or implant-retained zirconia restorations:* #**1**—((dental crowns [MeSH]) OR (full crown) OR (partial crown) OR (table top)) #**2**—((zirconia [MeSH]) OR (ZrO2) OR (zirconium dioxide))
	**Intervention**	*Speed sintered zirconia restoration* #**3**—((speed sintering) OR (high-speed sintering))
	**Comparison**	*Conventionally sintered zirconia restorations* #**4**—((sintering) OR (firing))
	**Outcome**	*Mechanical, clinical and economic outcomes including pre- and postprocessing* #**5**—((mechanical properties) OR (mechanical stability) OR (flexural strength) OR (microstructure) OR (grain size) OR (translucency)) #**6**—((volume stability) OR (internal fit) OR (marginal fit)) #**7**—((clinical behavior) OR (adverse event) OR (esthetics)) #**8**—((economics [MeSH Terms]) OR (time-efficiency) OR (cost effectiveness) OR (cost analysis))
	**Search combination(s)**	**(#1) AND (#2) AND (#3 or #4) AND (#5 or #6 or #7 or #8)**

**Table 2:** Characteristics of included studies. CS: Conventional sintering; S: Speed sintering SS: Super Speed sintering.

Author/ Publication Year	Journal	Study Type	Restoration [*n*]	Material/ Brand	Grain	Manufacturing Technique	Sintering (Sintering, Holding Time, Cooling Time)	Test Method	Control Group	Test Group	Outcome
Soult et al., 2019 [15]	Gen Dent	In vitro	20	CAD/CAM blocks (CEREC Zirconia, medi S, shade A2)	3-YTZP 0.2 to 0.8 μm	Milling unit (CEREC MC XL)	(1) high-speed furnace (CEREC SpeedFire furnace) for 26.2 min with pre-drying and proprietary firing parameters (2) conventional furnace (Programat S1 1600, Ivoclar Vivadent) for 4.3 h, dwell time: 2 h at 1510 °C	Fracture strength and modulus (3-point bending) Translucency and opalescence (spectrophotometer)	Sintering protocol 2	Sintering protocol 1	(1) No difference in flexural strength, flexural modulus, translucency or opalescence. (2) Smaller grain size after high-speed sintering 0.3 μm compared to conventional sintering 0.5 μm
Ahmed et al., 2019 [16]	J Proshtodont	In vitro	120	IPS e.max ZirCAD LT zirconia blanks (Ivoclar Vivadent US, Amherst NY)	3- YTZP	5-axis milling machine (Wield Select; IvoclarVivadent, Schaan, Liechtenstein)	1- Conventional sintering furnace (Programat S1 Furnace; Ivoclar Vivadent US). CS: 9 h 50 min2- S: 2 h 55 min	Marginal Discrepancy (Digital Microscope)	1–6: sintering protocol 1, 7–12: sintering protocol 2 G1/G7 (0.5 mm chamfer, 0.8 mm thick); G2/G8 (0.5 mm chamfer, 1.5 mm thick); G3/G9 (1.0 mm chamfer, 0.8 mm thick); G4/10 (1.0 mm chamfer, 1.5 mm thick); G5/G11 (1.2 mm chamfer, 0.8 mm thick); G6/G12 (1.2 mm cham- fer, 1.5 mm thick).		Significant interaction between finish line widths, crown thickness and sintering protocol on the marginal gaps in both sintering protocols
Nakamura et al., 2020 [17]	J Proshtodont Res	In vitro	28	InCoris TZI, Dentsply Sirona and (CEREC inLab MC X5, Dentsply Sirona)	3-YTZP	Milling machine (CEREC MC XL, Dentsply Sirona)	CS: at 1510 °C in laboratory furnace for 220 min (inFire HTC Speed, Dentsply Sirona) SS: at 1580 °C in laboratory furnace for 15 min (CEREC SpeedFire, Dentsply Sirona)	Fit (Digital microscope)Fracture load (Universal testing machine) Aging resistance (X-ray diffractometer)	Sintering protocol 1	Sintering protocol 2	Monolithic zirconia crowns produced by high-speed sintering showed no significant difference in the marginal gap and the fracture load after aging or occurrence of monoclinic crystals compared to conventional sintering.
Edwards Rezende et al., 2017 [18]	Dent Mater	In vitro	36	(1) Z-MAX, IPS e.max ZirCAD (Ivoclar Vivadent, Liechtenstein); (2) ZYZ, InCeram YZ (Vita Zahnfabrik, Germany); (3) ZK, Zirklein (Zirklein, Brazil)).	3-YTZP	Milling procedure (MC XL milling machine; Sirona Dental Systems GmbH—Bensheim, Germany)	High-temperature furnace was used (Sintramat High Temperature Furnace; Ivoclar Vivadent; Liechtenstein) with a default cycle of 7 h and 52 min and a maximum temperature of 1500 °C.	Marginal and internal fit (micro-CT) Dimensional change, sintering shrinkage rate (micro-CT)	Before sintering	After sintering	No difference for marginal fit, with differences only for internal fit and between the different regions measured. The lack of uniformity of sintering shrinkage might lead to a non-uniform internal fit of Y-TZP copings.
Wiedenmann et al., 2020 [19]	Dent Mater	In vitro	192	Ceramill Zolid HT+, Amann Girrbach AG)	3-YTZP 4-YTZP	Milling machine (Ceramill motion, Amann Girrbach AG)	Sintered at 1580 °C (high-speed sintering) or 1450 °C (control group	Fracture load with and without aging (Universal testing machine) Two body wear (3D laser scanner)	Sintering protocol 2	Sintering protocol 1; 3 different groups with different layers (0.5, 1, 1.5 mm)	High-speed sintering resulted in less two-body wear of the zirconia and comparable or even higher fracture load results than the control group.
Kaizer et al., 2017 [20]	Ceram Int	In vitro	30	translucent Y-TZP (inCoris TZI, Sirona)	3-YTZP	CAD/CAM-milled, sintered and glazed by Sirona	Super-speed (SS, 1580 °C, dwell time 10 min), Speed (S, 1510 °C, dwell time 25 min), and Long-term (LT, 1510 °C, dwell time 120 min).	- Microstructure (wear depth and volume loss for the steatite antagonists on 3D images of micro computed tomography scanner) - Wear (sliding wear testing using a chewing simulator) - Translucency (colorimeter (SpectroShadeTMMicro, MHT Optic Research AG, Switzerland)	3 Sintering protocols		Micropits in the wear crater were less frequent for the LT group. Groups S and SS exhibited more surface pits, scratched steatite surface and a greater volume loss. Tetragonal to monoclinic phase transformation, resulting from the sliding wear process, was present in all three groups.
Cokic et al., 2020 [21]	Dent Mater	In vitro	64	Katana STMLCS, STMLSS (Kuraray Noritake) CEREC Zirconia (CEREC ZrS) (Dentsply Sirona) inCoris TZICS	5-YTZP 3-YTZP 3-YTZP	Plates with dimensions of approximate 15 × 15 × 3.5 mm	Katana STMLSS (Kuraray Noritake) (total thermal cycle/sintering time/dwell temperature: 30 min/16 min/1560 °C) and CEREC Zirconia (CEREC ZrSS) (Dentsply Sirona) (15 min/2 min/1578 °C) were compared to conventionally sintered (CS) Katana STMLCS (6.8 h/2 h/1550 °C) and inCoris TZICS (4 h/2 h/1510 °C).	Translucency parameter and contrast ratio (spectrophotometer) Chemical composition (X-ray fluorescence spectroscopy) and phase composition (X-ray diffraction). Hydrothermal aging behavior (XRD) The indentation fracture toughness, Vickers hardness and biaxial strength of the sintered ceramics were assessed (Vickers micro-hardness tester, Universal testing machine).	2 Groups (CS)	2 Groups (S)	SS and CS zirconia revealed similar density, microstructure, average strength and hydrothermal aging stability. Both Katana STMLSS/CS 5Y-PSZ ceramics were characterized with a higher content of cubic phase (≈53 wt%), resulting in higher amount of Y_2_O_3_ in the remaining tetragonal ZrO_2_ phases compared to the 3Y-TZP CEREC ZrSS and inCoris TZICS (8 and 20 wt%, respectively). The sintering program did not affect the hydrothermal aging behavior of Katana STMLSS and CEREC ZrSS. TP of Katana STMLSS (TP ≈ 32) was not affected by speed sintering, while the translucency of CEREC ZrSS (TP = 14) was significantly reduced. Hardness, fracture toughness and Weibull characteristic strength of Katana STMLSS and CEREC ZrSS also reached the optimal level, but speed sintering substantially lowered their mechanical reliability.
Al-Zordk et al., 2020 [22]	J Prosthet Dent	In vitro	80	4 zirconia brands (Zolid FX Preshaded, Zolid FX White, DD Cubex2, and DD Bio ZX2)	3-Y-TZP	Milled from A2 pre-shaded blanks except for Zolid FX White disks which were milled from white blanks with subsequent immersion in A2 coloring liquid	Sintering time/dwell temperature: Zolid FX (120 min/1450 °C)Zolid FX White (120 min/1450 °C) DD Cubex2 (60 min/1450 °C)DD Bio ZX2 (50 min/1450 °C)	Color stability, contrast ratio and the translucency parameter after coffee thermocycling (reflectance spectrophotometer)	4 Groups (CS)	4 Groups (S)	The color and the translucency of the translucent zirconia can be affected by the type of the zirconia brand and the sintering protocol. Furthermore, the color and the translucency were affected by both the clinical adjustment procedure and the coffee thermocycling, but not beyond the clinically acceptable limit of the color difference.
Lawson et al., 2020 [23]	J Esthet Restor Dent	In vitro	40	(Katana STML Block, Prettau Ante- rior, and Zpex Smile) lithium disilicate material (IPS e.max CAD)	3-YTZP 5-YTZP 5-YTZP	Katana STML Block and Prettau Anterior (dry sectioned and dry polished). Specimens of Zpex Smile were fabricated by obtaining pure powders and pressing into molds. IPS e.max CAD (wet sectioned and wet polished)	Conventional (7 h) or high-speed (18 or 30 min in a SpeedFire furnace) sintering	- Translucency (Color-i7spectrophotometer); - Flexural strength (3-point bending test) - Grain structure (SEM)	lithium disilicate material	Katana STML, Prettau Anterior, and Zpex Smile	- Significant differences between materials for flexural strength, translucency parameter and grain size (*p* < 0.001). Grains became significantly larger, and pores were present when two of the zirconia materials (Prettau Anterior and Zpex Smile) were sintered with a high-speed sintering program. - Two of the zirconia materials (Prettau Anterior and Zpex Smile) became less translucent and less strong using a high-speed sintering program, whereas another (Katana STML Block) was unaffected.
Jansen et al., 2019 [24]	J Prosthet Dent	In vitro	450	Ceramill ZI Zolid (ZD) Zolid HT+	3-YTZP3-YTZP4-YTZP	5 thicknesses (1.0, 1.5, 2.0, 2.5, and 3.0 mm); milled (Ceramill Motion 2; Amann Girrbach AG)	Final temperature 1570 °C and 1590 °C and a reference sintering protocol (1450 °C)	monoclinic phase content (Raman spectrometry) translucency (UV-Vis spectrophotometer) Biaxial flexural strength (Universal testing machine)	Conventional sintering protocol	Speed sintering protocol	For ZI, the sintering protocols did not affect the translucency or biaxial flexural strength. ZD and HT+ showed significantly lower translucency for high-speed sintering protocols (*p* 0.001), but the biaxial flexural strength remained the same after the high-speed sintering protocol at 1590 °C. Grain sizes increased with increasing final sintering temperature for ZI and HT+, whereas translucency generally decreased with increasing material thickness. No monoclinic phase was detected in any group. The flexural strength was maintained with high-speed sintering but led to a decrease in translucency for ZD and HT+.
Lümkemann et al., 2021 [25]	J Prosthet Dent	In vitro	210	3Y-TZP0.25 (*n* = 30), 3Y-TZP0.05 (*n* = 30), 5Y-TZP (*n* = 30), 4Y-TZP (*n* = 60), pre4Y-TZP (preshaded, *n* = 30), and LiSi2 (*n* = 30)	3Y-TZP 3Y-TZP 5Y-TZP 4Y-TZP 4Y-TZP	Milled (Ceramill Match 2/Ceramill Motion 2; Amann Girrbach AG)	Conventionally sintered at 1450 °C (3Y-TZP0.25, 3Y-TZP0.05, 5Y-TZP, and half of 4Y-TZP) or high-speed sintered at 1580 °C (the other half of 4Y-TZP and pre4Y-TZP)	Translucency (UV/Vis spectrophotometer) flexural strength (Universal testing machine)	Conventional sintering protocol	Speed sintering protocol	The decrease in translucency related to aging hours was higher for LiS2 and conventional sintered zirconia materials than for 4Y-TZPspeed and pre4Y-TZPspeed. Initially, 3Y-TZP0.25 had the highest flexural strength, followed by 3Y-TZP0.05, 4Y-TZP and pre4Y-TZPspeed. pre4Y-TZPspeed was comparable with 4Y-TZPspeed but significantly higher than 5Y-TZP. LiSi2 had the lowest biaxial flexural strength. Hydrothermal aging increased biaxial flexural strength for 3Y-TZP0.25 and 3Y-TZP0.05 but decreased it for 5Y-TZP and pre4Y-TZPspeed. After aging, 4Y-TZPspeed showed comparable values of flexural strength with 4Y-TZP and higher values than pre4Y-TZPspeed after aging. Manually colored, conventionally sintered 4Y-TZP was resistant to hydrothermal aging regarding flexural strength. High-speed sintering inhibited color development for manually colored 4Y-TZP but did not affect the resistance to hydrothermal aging. The findings were reversed for industrially pre-shaded 4Y-TZP.
Jerman et al., 2020 [26]	Dent Mater	In vitro	288	(ZI Zolid; Zolid HT+; Amann Girrbach AG)	3-YTZP4-YTZP	Milled using a five-axis milling machine (Ceramill Motion 2, Amann Girrbach, Koblach, Austria)	High-speed sintering protocol (final temperature 1580 °C) or a conventional sintering protocol (control group, final temperature 1450 °C)	flexural strength (Universal testing machine)	Conventional sintering protocol	Speed sintering protocol	ZI showed the highest and HT+ the lowest FS, regardless of the sintering protocols and aging regimens. High-speed sintered HT+ showed higher initial FS than the control group. ZI and Zolid showed higher FS after thermo-mechanical aging. High-speed sintered HT+ showed higher FS in the initial stage.
Elisa Kauling et al., 2020 [27]	J Prosthet Dent	In vitro	48	zirconia blocks (CEREC Zirconia Medi S A2, Lot 2016219456; Dentsply Sirona),	3-YTZP	3-unit FDPSs milled (MCXL Premium; CEREC Zirconia; Dentsply Sirona)	Speed sintering (group S) by using the SpeedFire (Dentsply Sirona) for 25 min and the conventional sintering (group C) by using the inFire HTC speed (Dentsply Sirona) for 4 h	Fit (image analysis software pro- gram (Optimas 6.5 version 6.51–1999; Media Cybernetics) Fracture load (Universal testing machine)	Conventional sintering protocol	Speed sintering protocol	- Group S showed a better marginal and occlusal fit than group C. For the fracture load values, no significant difference was found because of the sintering procedure or the interaction of the sintering procedure and artificial aging. - Speed-sintered FPDs had equal and better values for the fit and fracture load than conventional sintering.
Michailova et al., 2020 [28]	J Mech Behav Biomed Mater	In vitro	96	Katana Zirconia STML Block (KZC), Katana Zirconia STML Disc (KZL) and IPS e. max ZirCAD Prime (EZL). Lithium disilicate ceramic (IPS e. max Press, ELC)	KZC and KZL: 4Y-TZP EZL: 3&5Y-TZP	Milled (Ceramill Motion2, Amann Girrbach, Koblach, Austria) milled (CEREC MCXL, Dentsply Sirona)	Sintered according to the manufacturer’s instructions (Nabertherm, Lilienthal, Germany) at 1550 °C (KZL) and 1500 °C (EZL). KZC crowns were high-speed sintered at 1560 °C for 19 min (CEREC SpeedFire, Dentsply Sirona). ELC crowns were crystallized according to the manufacturer instructions at 840 °C (Programat EP 5000, Ivoclar Vivadent, Schaan, Liechtenstein).	Translucency (UV/Vis spectrophotometer) Fracture load (Universal testing machine)	(ELC).	KZL, KZC, EZL	The high-speed sintering of zirconia showed neither a negative impact on the fracture load nor on the two-body wear. However, the optical properties and the reliability of zirconia is lower than those of highly translucent lithium disilicate ceramic.
Ersoy et al., 2015 [29]	Acta Biomater Odontol Scand	In vitro	120	(In-Coris ZI, In-Coris TZI)	3 Y-TZP	Cut using a low-speed diamond saw	Three groups and sintered at different final sintering temperatures and for various durations: CS) 1510 ° C for 120 min, S) 1540 °C for 25 min and SS) 1580 °C for 10 min.	Grain sizes (scanning electron microscopy (SEM)) phase transitions (X-ray diffraction (XRD)) Flexural strength (Universal testing machine)	Conventional sintering protocol	Speed sintering protocol	The highest flexural strength was observed in ZI and TZI samples sintered (SS). The differences between the ZI samples sintered at CS and those sintered at S were statistically insignificant. Also, TZI samples sintered at CS and those sintered at S also did not show any statistically significant differences. There were no visible differences in the grain sizes between the ZI and TZI specimens. The XRD patterns indicated similar crystalline structure for both materials subjected to the three different procedures. The results of this study showed that experimented high sintering temperature and short sintering time combination increases the flexural strength of zirconia.

## References

[1] Kinane D.F., Stathopoulou P.G., Paapanou P.N. (2017). Periodontal diseases. Nat. Rev. Dis. Primers..

[2] Suarez M.J., Perez C., Pelaez J., Lopez-Suarez C., Gonzalo E. (2018). A randomized clinical trial comparing zirconia and metal-ceramic three-unit posterior fixed partial dentures: A 5-year follow-up. J. Prosthodont..

[3] Bapat R.A., Yang H.J., Chaubal T.C., Dharmadhikari S., Abdulla A.M., Arora S., Rawal S., Kesharwani P. (2022). Review on synthesis, properties and multifarious therapeutic applications of nanostructured zirconia in dentistry. RSC Adv..

[4] Zhang F., Inokoshi M., Batuk M., Hadermann J., Naert I., Meerbeek B.V., Vleugels J. (2016). Strength, toughness and aging stability of highly-translucent Y-TZP ceramics for dental restorations. Dent. Mater..

[5] Alfawaz Y. (2016). Zirconia crown as single unit tooth restoration: A literature review. JCPD.

[6] Sailer I., Balmer M., Husler J., Hammerle C.H.F., Kanel S., Thoma D.S. (2018). 10-year randomized trial (RCT) of zirconia-ceramic and metal-ceramic fixed dental prostheses. J. Dent..

[7] Kern M., Passia N., Sasse M., Yazigi C. (2017). Ten-year outcome of zirconia ceramic cantilever resin-bonded fixed dental prostheses and the influence of the reasons for missing incisors. J. Dent..

[8] Baroudi K., Ibraheem S.N. (2015). Assessment of chair-side computer-aided design and computer-aided manufacturing restorations: A review of the literature. J. Int. Oral. Health.

[9] Roggendorf M.J., Kunzi B., Ebert J., Roggendorf H.C., Frankenberger R., Reich S.M. (2012). Seven-year clinical performance of CEREC-2 all-ceramic CAD/CAM restorations placed within deeply destroyed teeth. Clin. Oral. Investig..

[10] Alghazzawi T.F. (2016). Advancements in CAD/CAM technology: Options for practical implementation. J. Prosthodont. Res..

[11] Liu L.Y., Guo J.J., Du X.Y., Wang Q., Qiu L.H. (2019). Comparison of mechanical properties of three machinable resin ceramic composite materials. Shanghai Kou Qiang Yi Xue.

[12] Sulaiman T.A., Abdulmajeed A.A., Donovan T.E., Vallittu P.K., Nähri T.O., Lassila L.V. (2015). The effect of staining and vacuum sintering on optical and mechanical properties of partially and fully stabilized monolithic zirconia. Dent. Mater. J..

[13] Maginnis S., Carden R., Szeremeta A., Paskalov G. (2014). Method of Rapid Sintering of Ceramics. US Patent.

[14] Kim S.W., Khalil K.A.R. (2006). High-frequency induction heat sintering of mechanically alloyed alumina-yttria-stabilized zirconia nano-bioceramics. J. Am. Ceram. Soc..

[15] Soult M.D., Lien W., Savett D.A., Gallardo F.F., Vandewalle K.S. (2019). Effect of high-speed sintering on the properties of a zirconia material. Gen Dent..

[16] Ahmed W.M., Abdallah M.N., McCullagh A.P., Wyatt C.C.L., Troczynski T., Carvalho R.M. (2019). Marginal Discrepancies of Monolithic Zirconia Crowns: The Influence of Preparation Designs and Sintering Techniques. J. Prosthodont..

[17] Nakamura T., Nakano Y., Usami H., Okamura S., Wakabayashi K., Yatani H. (2020). In vitro investigation of fracture load and aging resistance of high-speed sintered monolithic tooth-borne zirconia crowns. J. Prosthodont. Res..

[18] Edwards Rezende C.E., Sanches Borges A.F., Macedo R.M., Rubo J.H., Griggs J.A. (2017). Dimensional changes from the sintering process and fit of Y-TZP copings: Micro-CT analysis. Dent. Mater..

[19] Wiedenmann F., Pfefferle R., Reichert A., Jerman E., Stawarczyk B. (2020). Impact of high-speed sintering, layer thickness and artificial aging on the fracture load and two-body wear of zirconia crowns. Dent. Mater..

[20] Kaizer M.R., Gierthmuehlen P.C., Dos Santos M.B., Cava S.S., Zhang Y. (2017). Speed sintering translucent zirconia for chairside one-visit dental restorations: Optical, mechanical, and wear characteristics. Ceram. Int..

[21] Cokic S.M., Vleugels J., Van Meerbeek B., Camargo B., Willems E., Li M., Zhang F. (2020). Mechanical properties, aging stability and translucency of speed-sintered zirconia for chairside restorations. Dent. Mater..

[22] Al-Zordk W., Saker S. (2020). Impact of sintering procedure and clinical adjustment on color stability and translucency of translucent zirconia. J. Prosthet. Dent..

[23] Lawson N.C., Maharishi A. (2020). Strength and translucency of zirconia after high-speed sintering. J. Esthet. Restor. Dent..

[24] Jansen J.U., Lümkemann N., Letz I., Pfefferle R., Sener B., Stawarczyk B. (2019). Impact of high-speed sintering on translucency, phase content, grain sizes, and flexural strength of 3Y-TZP and 4Y-TZP zirconia materials. J. Prosthet. Dent..

[25] Lümkemann N., Stawarczyk B. (2021). Impact of hydrothermal aging on the light transmittance and flexural strength of colored yttria-stabilized zirconia materials of different formulations. J. Prosthet. Dent..

[26] Jerman E., Wiedenmann F., Eichberger M., Reichert A., Stawarczyk B. (2020). Effect of high-speed sintering on the flexural strength of hydrothermal and thermo-mechanically aged zirconia materials. Dent. Mater..

[27] Elisa Kauling A., Güth J.F., Erdelt K., Edelhoff D., Keul C. (2020). Influence of speed sintering on the fit and fracture strength of 3-unit monolithic zirconia fixed partial dentures. J. Prosthet. Dent..

[28] Michailova M., Elsayed A., Fabel G., Edelhoff D., Zylla I.M., Stawarczyk B. (2020). Comparison between novel strength-gradient and color-gradient multilayered zirconia using conventional and high-speed sintering. J. Mech. Behav. Biomed. Mater..

[29] Ersoy N.M., Aydogdu H.M., Degirmenci B.Ü, Çökök N., Sevimay M. (2015). The effects of sintering temperature and duration on the flexural strength and grain size of zirconia. Acta. Biomater. Odontol. Scand..

[30] Liu H., Inokoshi M., Nozaki K., Shimizubata M., Nakai H., Cho Too T.D., Minakuchi S. (2022). Influence of high-speed sintering protocols on translucency, mechanical properties, microstructure, crystallography, and low-temperature degradation of highly translucent zriconia. Dent. Mater..

[31] Gintaute A., Weber K., Zitzmann N.U., Brägger U., Ferrari M., Joda T. (2021). A Double-Blind Crossover RCT Analyzing Technical and Clinical Performance of Monolithic ZrO_2_Implant Fixed Dental Prostheses (iFDP) in Three Different Digital Workflows. J. Clin. Med..

